# Hypoxia-inducible factor-1α induces CX3CR1 expression and promotes the epithelial to mesenchymal transition (EMT) in ovarian cancer cells

**DOI:** 10.1186/s13048-019-0517-1

**Published:** 2019-05-10

**Authors:** Santosh Kumar Singh, Manoj Kumar Mishra, Rajesh Singh

**Affiliations:** 1 Department of Microbiology, Biochemistry and Immunology, Morehouse School of Medicine, 720 Westview Drive SW, Atlanta, GA USA; 2 Department of Biological Sciences, Alabama State University, 915 S Jackson Street, Montgomery, AL USA

**Keywords:** Ovarian cancer, Hypoxia, Chemokines, CX3CR1, EMT markers

## Abstract

**Background:**

Chemokines are involved in the homing of various cancer cells, including those of ovarian cancer (OvCa), to distant organs. They may also promote or inhibit cancer progression and metastasis. Hypoxia, a common phenomenon in malignant tumors, promotes cell proliferation regulated by HIF-1α. Hypoxia-induced genes are involved in metastasis-associated functions and in the epithelial-to-mesenchymal transition (EMT).

**Results:**

Tissue microarrays of human OvCa showed elevated expression of CX3CR1 and HIF-1α compared to normal cells, and their levels were higher in adenocarcinoma stages II and III. To substantiate these observations, we performed studies using OvCa cells. Following exposure to hypoxia, OVCAR-3, SW 626, and TOV-112D cells showed high expression of CX3CR1, a transmembrane protein involved in the adhesion and migration of leukocytes, causing an increased chemotactic response to CX3CL1, the ligand for CX3CR1. As determined by flow cytometry, immunofluorescence, RT-PCR, and western blots, there were higher expressions of CX3CR1 and HIF-1α in OvCa cell lines exposed to hypoxia. Further, OvCa cells expressing CX3CR1 were sensitive to the CX3CL1 ligand. Chemotaxis based on chemokine receptors was influential in elevating the expression of EMT markers and matrix metalloproteinases, which are involved in the progression and metastasis of cancer cells.

**Conclusions:**

In OvCa cells, CX3CR1 was upregulated in a process involving hypoxia-mediated regulation of HIF-1α. The elevated levels of CX3CR1, which were sensitive to CX3CL1, increased EMT markers that led to the progression and metastasis of OvCa. Thus, CX3CR1 and HIF-1α are suitable targets for treatment of OvCa.

## Background

Ovarian cancer (OvCa), the deadliest gynecological malignancy, is the seventh most commonly diagnosed cancer among women [1]. Although 90% of OvCas originate in the epithelium, the disease is heterogeneous, with histologic subtypes that differ in their cellular origin [2]. Several genes have been implicated in familial OvCa, and mutations in BRCA1 and 2 are associated with a higher risk of cancer development. In addition, alterations in vascular endothelial growth factor and the PI3K/AKT/mTOR pathway are implicated in OvCa [3].

Chemokines, first described as chemoattractant cytokines synthesized at sites of inflammation, are regulatory proteins for leukocyte recruitment and trafficking. Chemokines are subdivided into four families, C, CC, CXC, and CX3C, based on the number and spacing of the first two cysteines in a conserved cysteine structural motif. CX3CL1 (also known as fractalkine), the sole member of the CX3C class of chemokines, exists in soluble and membrane-anchored forms. The cognate receptor of CX3CL1 is a G-protein-coupled receptor, CX3CR1, a transmembrane protein involved in the adhesion and migration of leukocytes. Along with expression in certain leukocyte populations, such as macrophages, lymphocytes, and natural killer cells, CX3CR1 is abundant on glial cells and astrocytes and also in tumors. Among the chemokine receptors expressed by OvCa, CX3CR1 is expressed by primary OvCa cells and is activated by its ligand, CX3CL1 [4]. The role of this chemokine receptor-ligand (CX3CR1-CX3CL1) interaction in OvCa metastasis is substantiated by impairment of their interaction by antibodies and/or by shRNA raised against the CX3CL1 ligand [4].

Rapidly proliferating tumor cells may cause depletion of oxygen to non-physiological levels due to compression of blood vessels, reducing the flow of oxygenated blood to tumors, and making them hypoxic [5, 6]. In cancer cells, hypoxia causes genetic changes [7] that induce expression of hypoxia-inducible factor 1α (HIF-1α), a transcription factor that binds to hypoxia-response elements involved in angiogenesis and glucose metabolism, and in cell proliferation, invasion, and metastasis [7]. The pathophysiological response of cancer cells to hypoxia involves a complex signaling network, which allows cells to adapt to the low levels of oxygen [8]. These interactions and the altered metabolism of cancer cells mediate acquisition of the epithelial-to-mesenchymal transition (EMT) phenotype, leading to their migration to distant sites, a process described as metastasis [9].

Metastasis is a complex process in which tumor cells penetrate the primary membrane, survive in the bloodstream, and arrive at a secondary site [10]. This process requires a transition from the epithelial to a mesenchymal state of the tumor cells, which adopt a spindle-shaped morphology and develop migratory potential [11]. The EMT, which involves various signaling pathways, is regulated by a set of transcription factors, including Snail, Slug, and Twist, which are regulators of cancer metastasis. These factors lead to loss of cell-cell adhesion molecules, such as E-cadherin, and gain of mesenchymal proteins, such as vimentin [11].

The conventional view of OvCa pathogenesis is that a tumor undergoes progressive dedifferentiation to a poorly differentiated state, to infiltrating cancer, and subsequently metastasizing to distant sites. Metastatic progression of OvCa is associated with complex signal transduction mechanisms leading to shedding of the malignant cells from the primary tumor followed by their establishment at the organs of the peritoneal cavity, anchorage in the sub-mesothelial extracellular matrix, and establishment of metastases [12]. The signal transduction involved in metastasis is partly regulated by transmembrane domain receptors such as the chemokine receptors activated by secreted protein-ligand chemokines [13]. The chemokine receptors expressed by OvCa cells, and their interaction with chemokine ligands mediate the metastasis in OvCas [13].

In OvCas, hypoxic conditions regulate the expression of chemokine ligands, CXCL12 and CCL28 [14], and the chemokine receptors, CCR2, CXCR1, CXCR2, CXCR4 14 [5]. In prostate cancer cells, hypoxia regulates CXCR6 and CX3CR1, which are involved in migration and invasion [5, 15]. However, whether hypoxia is involved in the metastasis of OvCa cells through regulating CX3CR1 expression has not been elucidated. In the present investigation, we determined the role of hypoxia in promoting the expression of CXC3R1 in OvCa cells and increasing their potential for migration and invasion.

## Results

### CX3CR1 and HIF-1α expression by OvCa tissues

**Fig. 1:**
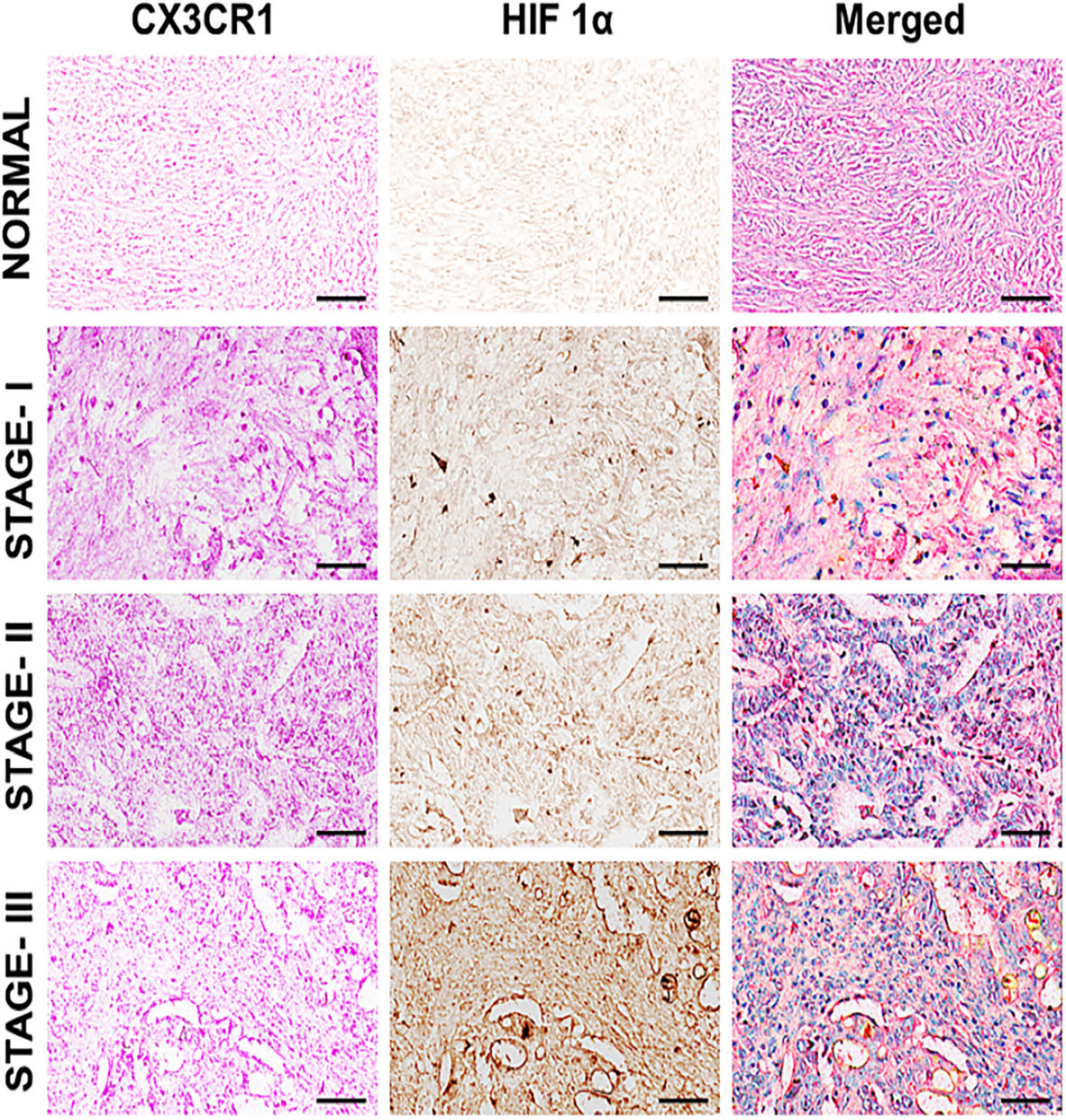
CX3CR1 and HIF-1α expression by OvCa tissues: Ovarian tissues from normal and various cancer stages [well-differentiated (Stage I), moderately differentiated (Stage II), and poorly differentiated (Stage III)] were stained with anti-CX3CR1 and anti-HIF-1α antibodies. Magenta (AP) color shows CX3CR1, and Brown (DAB) color shows HIF-1α staining. An Aperio ScanScope CS system with a 40X objective captured digital images of each tissue. Representative cases are immuno-intensities of CX3CR1 and HIF-1α using image analysis Aperio ImageScope v.6.25 software

Tumor hypoxia is a problem in anticancer therapy, and the interactions of chemokines and their receptors determine the metastatic destinations of tumor cells [16]. To establish the role of CX3CR1 and hypoxia in OvCa progression and metastasis, we performed TMAs of 75 human OvCas, which were stained and analyzed for CX3CR1 and hypoxia-inducible factor 1α (HIF-1α). The expressions of CX3CR1 and HIF-1α were particularly high in adenocarcinomas of stages II and III, compared to normal OvCa tissues (Fig. 1). These observations correlate with previous findings in which HIF-1 and CX3CR1 are associated with tumor metastasis [16]. However, their function in OvCa progression remains elusive. Our results showed that, among adenocarcinomas, stage III had higher expressions of CX3CR1 in the membrane and cytoplasm. However, a lack of expression of HIF-1α and minimal expression of CX3CR1 were evident in normal OvCa tissues, indicating that the CX3CR1 and HIF-1α interaction mediates OvCa.

### Expression of CX3CR1/ CX3CL1 by OvCa cells

**Fig. 2:**
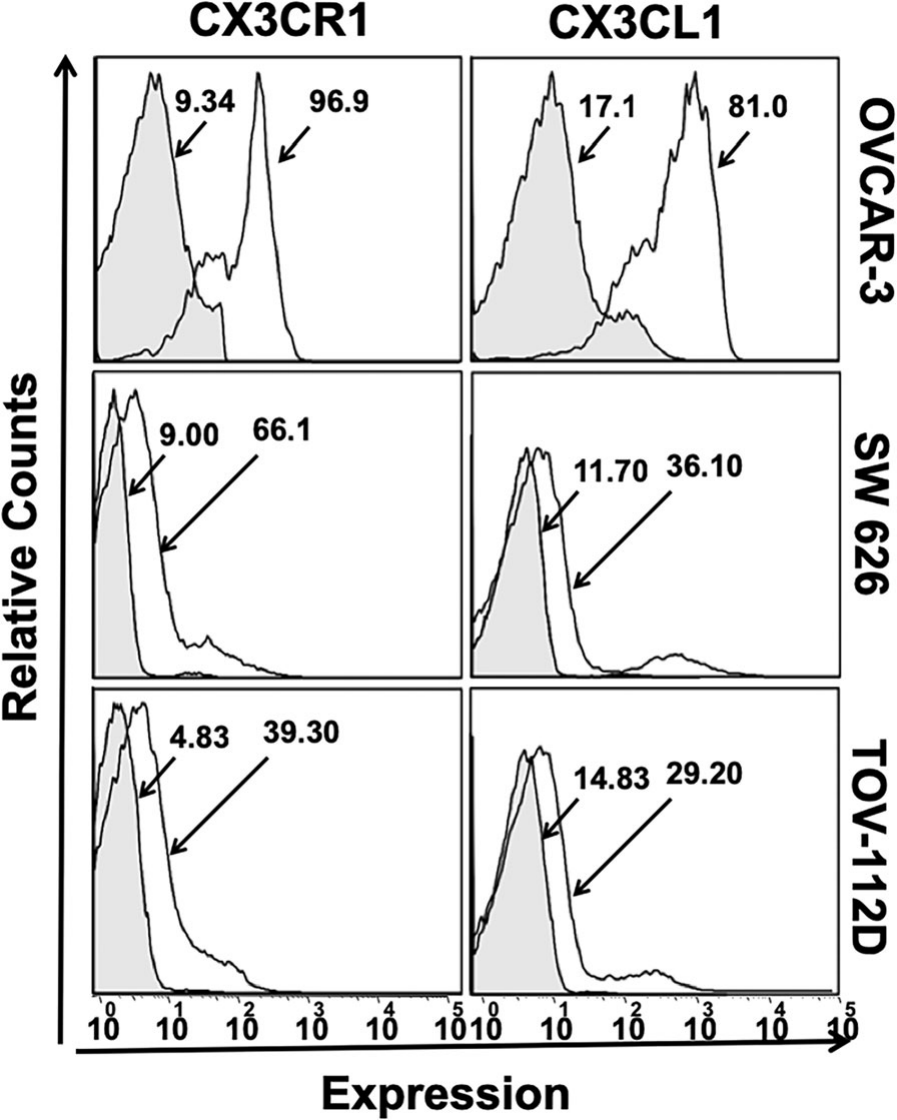
Expression of CX3CR1 and CX3CL1 by OvCa cells. OVCAR-3, SW 626, and TOV-112D cells were stained with FITC-conjugated or anti-CX3CR1 and PE-conjugated isotype or anti-CX3CL1 antibodies, and the expressions were quantified by flow cytometry. The gray and white histograms represent isotypes and CX3CR1/CX3CL1 fluorescence intensity, respectively

Furthermore, to evaluate the expressions of CX3CR1 and CX3CL1, we analyzed OVCAR-3, SW 626, and TOV-112D cells by flow cytometry. All cells were stained with FITC-conjugated isotype or anti-CX3CR1 and phycoerythrin (PE)-conjugated isotype or anti-CX3CL1 antibodies, and expression were measured in terms of mean fluorescent intensity. Among these cell lines, OVCAR-3 cells showed higher CX3CR1 expression compared to SW 626 and TOV-112D cells. The mean fluorescent intensities of CX3CR1 were 96.9, 66.1, and 39.30 in OVCAR-3, SW 626, and TOV-112D cells, respectively **(**Fig. 2**)**. Similarly, CX3CL1 expression was high in OVCAR-3 (81.0) compared to SW 626 (36.1) and TOV-112D (29.2) cells. These observations indicate that chemokine-receptor interaction based OvCa regulation.

### Cellular expression of HIF-1α in hypoxic OvCa cells

**Fig. 3:**
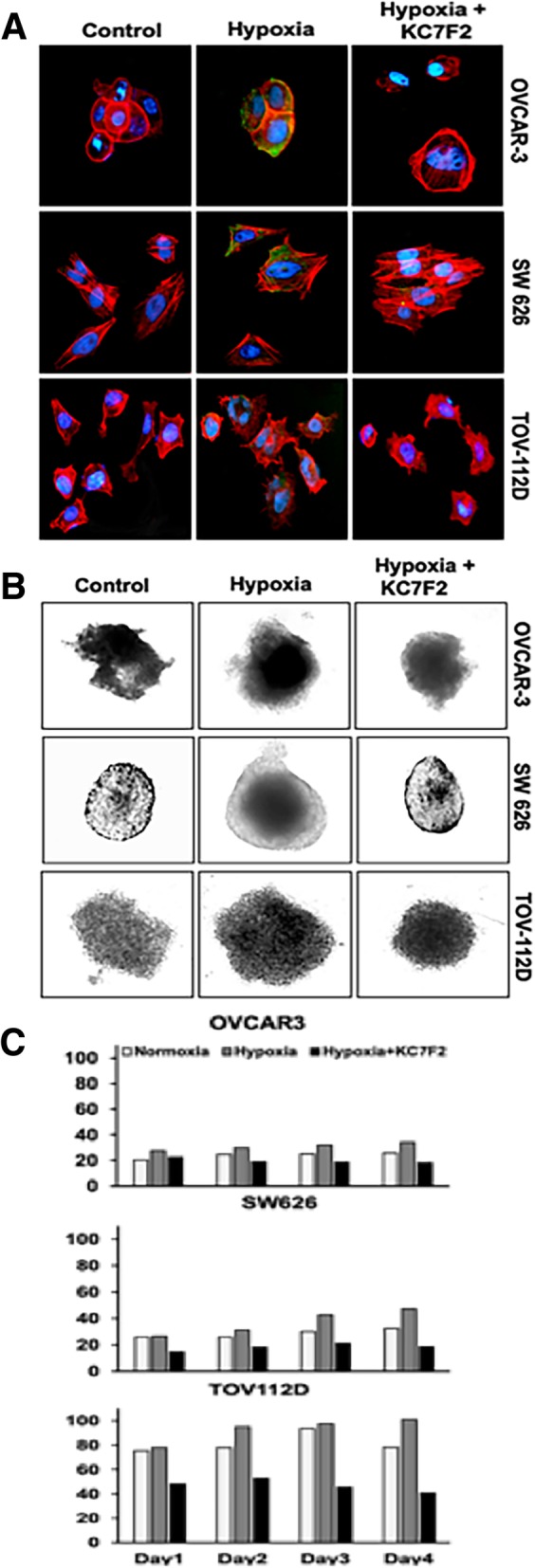
Cellular localization of HIF-1α and cell growth/proliferation under hypoxia for OVCAR-3, SW 626 and TOV-112D cells**.** (**a**) To determine the effect of hypoxia, the OvCa cells were treated with CoCl_2_ (150 μM), and with HIF-1α inhibitor KC7F2 for 12 h. Subsequently, cells were fixed and washed, followed by staining with primary (HIF-1α), and secondary antibody (Alexa Flor 488). Nuclei were counterstained with DAPI. Green fluorescence indicates HIF-1α, and red designates phalloidin. The stained cells were imaged with a 40X objective. (**b**) Morphology of 3D cell growth and proliferation with or without inhibitor under hypoxia. The inhibition by KC7F2 confirmed the role of hypoxia in OvCa cells. (**c**) Quantitative analysis for surface area of OvCa spheroids over a period of 4 days after treatment with or without inhibitor under hypoxia

To investigate the expression of HIF-1α in hypoxic cells, we analyzed the profile of HIF-1α in OvCa by fluorescence microscopy. Immunofluorescence (IF) staining showed elevated expression of HIF-1α (green) under hypoxic conditions compared to the normoxic state (Fig. 3a). Of the three cell lines, OVCAR-3 and SW 626 showed higher expression of HIF-1α compared to TOV-112D cells. To confirm that hypoxia is the most relevant factor involved in the expression of HIF-1α, the addition of KC7F2 (an inhibitor) to the medium along with CoCl_2_ reduced the expression of HIF-1α. Moreover, the addition of the inhibitor reversed the intensity of HIF-1α expression in cells, indicating that hypoxia mediates the proliferation of OvCa cells.

We also assessed the proliferation of the cell lines grown as 3D spheroids under hypoxic conditions in the presence and absence of the HIF-1α inhibitor. Cell growth was elevated in the hypoxic condition compared to normoxia **(**Fig. 3b & c**)**. However, in the presence of KC7F2, growth was reduced in hypoxic cells compared to normoxic cells. These results show that hypoxia regulates the growth of OvCa cells.

### **Hypoxia activates HIF-1**α **and its regulatory and EMT marker expression in OvCa cells**

**Fig. 4:**
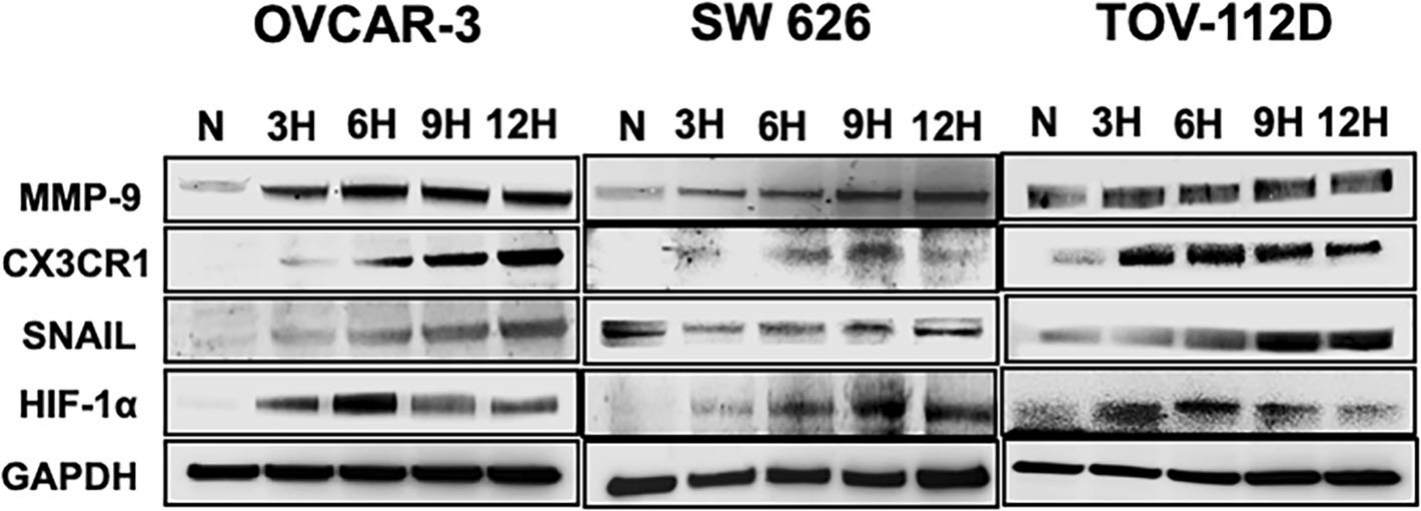
Western blot expression of hypoxia regulatory markers in OvCa cells. Immunoblot detection of OVCAR-3, SW 626, and TOV-112D cells exposed to 3, 6, 9, or 12 h hypoxia respectively. Immunoblotting was accomplished with primary antibodies against HIF-1α, CX3CR1, EMT marker (Snail), and MMP-9. As an internal standard for equal loading, anti-GAPDH antibody was used to probe blots

With cells under reduced oxygen tension, HIF-1α is translocated to the nucleus, where it activates transcriptional factors that in turn regulate tumor angiogenesis, migration, invasion, and metastasis [17]. To examine the role of HIF-1α as a regulator of a cellular response, immunoblot blot assays of OvCa cells were performed. Cells were exposed to 1% O_2_, 5% CO_2_, and 92% N_2_ for 3, 6, 9, or 12 h, and, for the hypoxia mimicking effect, CoCl_2_ (150 μM) was used. Western blot analysis revealed increased expression of HIF-1α when the OvCa cells were exposed to hypoxic conditions for 6 h (OVCAR-3 and TOV112D) or 9 h (SW 626) **(**Fig. 4**)**. However, for the three cell lines, there was a decrease of HIF-1α expression at 12 h of hypoxia. Thus, hypoxic cells, compared to normal cells, have elevated expression of the HIF-1α protein.

Next, we investigated the hypoxia-induced signaling molecules and their chemotactic response. The OvCa cell lines were placed under hypoxia to examine the expression of CX3CR1 **(**Fig. 4**)**. For all the three cell lines (OVCAR-3, SW 626, and TOV-112D), hypoxia-induced CX3CR1 upregulation. SW626 cells showed higher expression at 6 and 9 h. TOV112D cells showed enhanced expression at 3 and 6 h. For OVCAR-3 cells, CX3CR1 expression was elevated at 9 and 12 h. Furthermore, a hypoxia-induced EMT was characterized by an increase in mesenchymal-like marker, Snail. OVCAR-3, SW 626, and TOV-112D cells showed strong expression of Snail at 6, 9, and 12 h. In relation to angiogenesis, hypoxia enhanced MMP-9 expression in OVCAR-3 and SW 626 cells in a time-dependent manner. However, there was no significant difference in MMP-9 expression in TOV-112D cells. These results indicate that HIF-1α contributes to OvCa metastasis through regulation of expression of target genes.

### Hypoxia-induced EMT and mRNA expression of regulatory molecules in OvCa cells

**Fig. 5:**
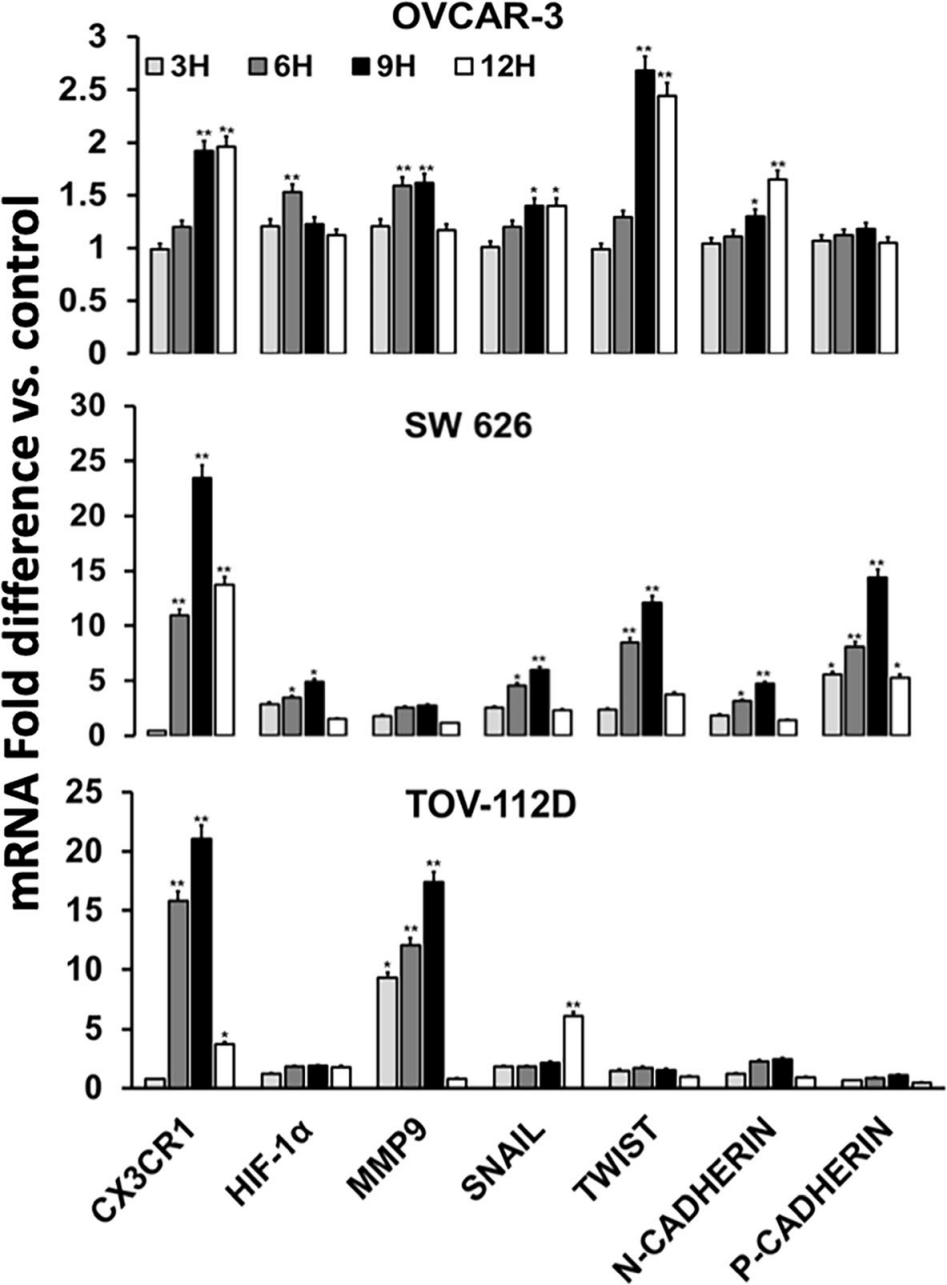
mRNA analysis of hypoxia-induced signaling molecules in OvCa cells. Ovarian tumor cells (OVCAR-3, SW 626, and TOV-112D) were exposed to hypoxia for 3, 6, 9, or 12 h. Quantitative RT-PCR results for expressions of CX3CR1, HIF-1α, MMP9, Snail, Twist, N- and P-cadherin are shown. The data were normalized to the levels of the housekeeping gene (18 S), and the analyses were accomplished in triplicate. Data were presented as fold change in expression (± standard error); the asterisks indicate significant differences as determined by Student’s *t*-test (*P < 0.05; **P < 0.01)

A brief exposure to hypoxia allows tumor cells to survive and progress by activating hypoxia-inducible pathways such as PI3K/AKT/mTOR, NFkB, ERK and chemokines, EMT, migration, and inflammation [8]. To confirm the transcriptional activity of HIF-1α, real-time PCR was used to analyze its mRNA expression levels in OvCa cells. Cells were exposed to 1% O_2_, 5% CO_2_, and 92% N_2_ 3, 6, 9, or 12 h, and mimic with CoCl_2_. After 6 and 9 h of exposure to hypoxia, CX3CR1 expression was higher in SW 626 and TOV-112D cells (Fig. 5). OVCAR-3 cells had increased expression at 9 and 12 h of exposure. Moreover, OVCAR-3 cells showed HIF-1α significant expression at 6 h and SW 626 cells after 6 h and 9 h of exposure. TOV-112D cells had marginally increased expression. Next to HIF-1α, expression of MMP9 in OVCAR-3 was higher at both 6 and 9 h with negligible difference while it was maximum at 9 h in TOV-112D cells. However, MMP9 expression was negligible in SW 626 like HIF-1α. In addition to HIF-1α and MMP-9, the expression of EMT markers, Snail, Twist, N-cadherin, and P-cadherin was analyzed and they were upregulated in OVCAR-3 (N-cadherin 9 and 12 h) and SW 626 cells after 9 h of exposure. In contrast, there was no significant difference in TOV-112D cells for TWIST, N-cadherin, or P-cadherin; however, Snail was increased at 12 h.

### The CX3CR1/CX3CL1 interaction induces migration and invasion of OvCa cells

**Fig. 6:**
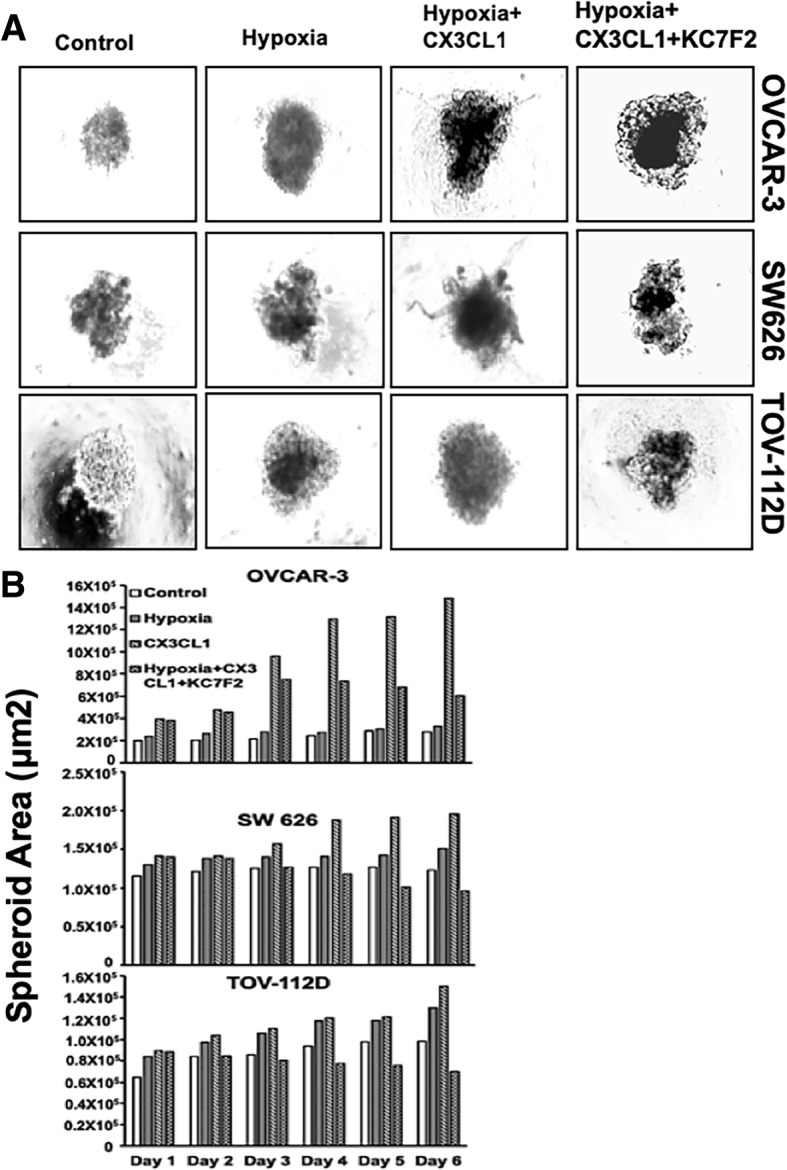
Migration and invasion induced by the CX3CR1/CX3CL1 interaction in hypoxic OvCa cells. OVCAR-3, SW 626, and TOV-112D cells were cultured in the presence of CoCl_2_ (150 μM) (to mimic hypoxia), and with or without CX3CL1 (chemoattractant) and KC7F2 (inhibitor) in the medium. Over 6 days, migration and invasion were assessed. (**a**) Morphology and (**b**) quantitative analysis of surface area, representing the invading cells projecting out of the spheroid into the medium when using CX3CL1. For cells under hypoxia, there was minimal growth, or they remained as aggregates when treated with or without the inhibitor, KC7F2

To investigate the role of the CX3CR1/CX3CL1 interaction leading to the migration and invasion of OvCa cells, we performed 3D spheroid cell invasion assays using OVCAR-3, SW 626, and TOV-112D cells. With the addition of the recombinant protein CX3CL1 to the medium, there were more OVCAR-3, SW 626, and TOV-112D cells projecting into the surrounding matrix compared to the control cells (without chemokine). This was also true for cells with hypoxia (mimicked with CoCl_2_) (Fig. 6a &b). To substantiate that cell migration was due to hypoxia, the function of HIF-1α was inhibited by KC7F2. The invasive capability of the OvCa cells was reduced, and the cells remained as aggregates. These findings indicated a correlation between HIF-1α expression and the migration and invasion of OvCa cells.

## Discussion

Hypoxia has numerous effects on cancers: their initiation and progression, dormancy, metabolism, and stemness are affected [18]. It influences the production of HIFs [18], which, in turn, activate genes involved in angiogenesis, glucose metabolism, cell proliferation/viability, invasion, and metastasis. HIFs have functions in tumor survival and progression [19]. In the present investigation, the expression of HIF-1α observed in samples of Stage II and Stage III OvCas was indicative of their invasiveness and metastatic nature.

Studies on the role of chemokine receptors, a family of proteins belonging to the group of G protein-coupled receptors, in OvCas place this family of membrane receptors as regulators of progression of malignancy [13]. The chemokine receptor CX3CR1 has a role in primary and metastatic OvCa cells [4], and hypoxia induces chemokine receptors such as CXCR4 and CX3CR1 [5, 8]. Based on these facts and the importance of CX3CR1 in OvCa, we stained tissues for CX3CR1 and found it to be elevated along with HIF-1α. Since HIF-1α could regulate the expression of CX3CR1 in OvCa cells, we analyzed the levels of CX3CR1 in the presence and absence of a HIF-1α inhibitor. Our studies with cultured cells, in which CX3CR1 expression was higher in hypoxic OVCAR-3, SW 626, and TOV-112D cells, substantiated the observations made with the clinical samples. The findings made with these cells also supported by the results for clinical samples, in which CX3CR1 and HIF-1α levels are elevated as cancers progress [16]. These results demonstrated the role of HIF-1α in regulating the expression of CX3CR1 in OvCa.

Hypoxic cells are more aggressive and invasive and have a better capacity to metastasize [20]. Hypoxia influences the invasive and migratory potential of cancer cells via the EMT, a trans-differentiation of cells in which they acquire plasticity and mobility [8]. The hypoxia-mediated EMT is characterized by lower expressions of epithelial markers (E-cadherin, β-catenin) and higher expressions of mesenchymal genes (N-cadherin, vimentin, and α-smooth muscle actin) [8]. Invasion of surrounding tissues is enhanced by HIF signaling through the upregulation and secretion of proteolytic enzymes, such as the matrix metalloproteinases (MMPs). In the present investigation, we found that hypoxia-regulated the expression of EMT markers and MMP-9, delineating the role of HIF-1α in the metastasis in OvCa.

Chemokines are involved in cancer progression and metastasis. The CXC3R1/CX3CL1 interaction is essential for the metastasis of cancer cells. CX3CL1, the solitary member of the CX3C, is expressed in a variety of cells, including neurons, dendrites, hematopoietic cells, endothelial cells, and epithelial cells [21–24]. Unlike other chemokines, CX3CL1 has unique features in that CX3CR1 is its only receptor, and it exists in membrane-bound and soluble forms [25]. The membrane-bound ligand is involved in adhesion and migration; the soluble form is involved in metastasis of tumors that express CX3CR1 [11, 21, 26]. In addition to hypoxia, for various cancers, growth and metastasis mediated by the chemokine/chemokine receptor axis are evident [5]. In breast cancer cells, CX3CL1 promotes the EMT by delocalizing E-cadherin, which enhances cancer cell motility [26]. In addition, in prostate cancer cells, CX3CL1 is involved in EGFR transactivation and expression of Slug [11]. Although we did not show a direct correlation between the CX3CR1/CX3CL1 axis and expression of EMT markers, the combined effect of HIF-1α and the CX3CR1/CX3CL1 axis appears to increase the metastatic potential of OvCa cells.

## Conclusions

Hypoxia, a characteristic of some solid cancers, bestows them with aggressiveness and resistance to drugs and promotes the metastasis of cancer cells. For various cancers, HIF-1α-regulated chemokine receptor-mediated metastasis of cancer cells has been elucidated. In the present study, we found that CX3CR1 was upregulated in hypoxic OvCa cells through HIF-1α, resulting in cell sensitivity to CX3CL1-stimulated migration and invasion. We illustrate that CX3CR1 is upregulated in OvCa in presence of hypoxia-mediated through HIF-1α. Further, the migration of OvCa cells was through elevated expression of EMT markers, such as Snail, Twist, N-cadherin, and P-cadherin. Thus, CX3CR1 and HIF-1α represent suitable targets for preventing and treating metastatic OvCa.

## Materials and methods

### Cell lines and hypoxic cultures

The human OvCa cell lines, OVCAR-3 (HTB-161), SW 626 (HTB-78), and TOV-112D (CRL-11731), were purchased from the American Tissue Culture Collection (ATCC, Manassas, VA). OVCAR-3 cells were grown in RPMI-1640 media supplemented with 0.01 mg/ml bovine insulin, and 20% fetal bovine serum (FBS) (Fisher Scientific, Pittsburgh, PA). SW 626 cells were grown in L-15 media (Fisher Scientific, Pittsburgh, PA) supplemented with 10% FBS. TOV-112D cells were maintained in a 1:1 mixture of MCDB 105 medium containing 1.5 g/L sodium bicarbonate and Medium 199 containing 2.2 g/L sodium bicarbonate, supplemented with 15% FBS and penicillin/streptomycin antibiotic solution (Fisher Scientific, Pittsburgh, PA). All cell lines were maintained in an incubator at 37 °C and 5% CO_2_.

For hypoxic exposure, cells were incubated in a humidified atmosphere of 1% O_2_, 5% CO_2_ and 94% N_2_ at 37 °C for 3–12 h in a special hypoxia environmental chamber (Stem Cell technology, Canada). Alternatively, for cells in a standard cell culture incubator, CoCl_2_ (150 μM) (Fisher Scientific, Pittsburgh, PA) was added to the medium to mimic a hypoxic environment.

### Immunohistochemistry

Tissue microarray (TMA) slides were procured from US BIOMAX, Inc. (Derwood, MD). The slides contained samples of 75 cases diagnosed with stage I (*n* = 48), stage II (*n* = 14), or stage III (*n* = 13). The method of TMA staining followed our previous publication [27]. Briefly, paraffin-embedded tissue sections were deparaffinized in xylene and rehydrated in a graded alcohol series (100, 95, and 70%, 5 min each). Antigen retrieval was performed to enhance the epitope availability by following a protocol for retrieving antigens (BioLegend, San Diego, CA). First, tissue was incubated for 10 min in the retrieving buffer at 92 °C, cooled at room temperature for 10 min, and rinsed with phosphate-buffered saline (PBS). Endogenous peroxidase was blocked by 3% H_2_O_2_. The slides were rinsed with PBST (PBS+ 0.05% Tween 20), blocked with 5% normal donkey serum for 1 h at room temperature, and then incubated with mouse anti-HIF-1α (1:40, Novus Biologicals, USA) antibody at 4 °C overnight or mouse anti-human CX3CR1 (1:50, R&D systems, USA) for 3 h at room temperature. After washing with PBS three times (5 min each), tissues were incubated with secondary antibody donkey anti-mouse antibodies (R&D Systems, Minneapolis, MN) for HIF-1α and CX3CR1 for 1 h at room temperature. After washing with PBS-T, tissue sections were incubated with streptavidin-horseradish peroxidase (HRP, Biolegend) (HIF-1α) or streptavidin-alkaline phosphatase (Jackson ImmunoResearch) (CX3CR1) and developed in diaminobenzidine and/or alkaline phosphatase red chromogen coloring agent. Then slides were counterstained with hematoxylin, dehydrated, and mounted. Digital images were captured and analyzed with an Aperio ScanScope scanning system (Aperio Technologies, USA).

### Flow cytometry

OvCa cells were grown overnight, trypsinized (0.05% trypsin), harvested, washed with PBS, and then counted with a hemocytometer (Countess II FL, Life Technology) as described previously [28]. To assess membrane staining, cells were exposed to 3% bovine serum albumin for 20 min at 4 °C to block non-specific binding sites and then incubated with FITC-conjugated isotype or anti-CX3CR1 and phycoerythrin (PE)-conjugated isotype or anti-CX3CL1 antibodies, cells were washed with FACS buffer supplemented with 2% FBS (Fisher Scientific, Pittsburgh, PA), fixed (4% paraformaldehyde), and permeabilized for 15 min with 0.05% saponin (Fisher Scientific, Pittsburgh, PA) prior to incubation with the CX3CL1 antibody. Subsequently, cells were washed and suspended in FACS buffer. Cells (5 × 10^4^) were analyzed in a flow cytometer using guava easyCyte HT (EMD Millipore, Billerica, MA).

### Immunofluorescence assays

OvCa cells (OVCAR-3, SW 626, and TOV-112D) were seeded in 48-well plates overnight and treated with CoCl_2_ (150 μM), to mimic hypoxia, and with KC7F2 (40 μM, Selleckchem, USA), an inhibitor of HIF-mediated transcription, for 12 h at 37 °C and under 5% CO_2._ Next, cells were fixed with 4% paraformaldehyde and permeabilized with 0.05% saponin for 10 min. Cells were washed and stained with and HIF-1α primary antibody (Invitrogen, USA) at 4 °C overnight, then incubated with FITC conjugated fluorescent secondary antibody (R&D System, USA) for 1 h at room temperature. Cells were washed with PBS to remove unbound antibody. To visualize the F-actin cytoskeleton, cells were stained with Phalloidin TM Red 594 solution (1:40) (BioLegend, USA) for 20 min at room temperature. Nuclei were counterstained with 4′,6-diamidino-2-phenylindole (DAPI, 300 nM, Invitrogen, USA) for 5 min. Images were captured by a fluorescent microscope with the appropriate filter for FITC/GFP, RFP, or DAPI with an EVOS FL microscope (Thermo Scientific, USA).

### Western blot analyses

To determine the expression of signaling molecules induced by HIF-1α in OvCa cells, the proteins were isolated following the method used in our previous publication [29]. First, OvCa cells exposed for 3 , 6 , 9 , and 12 h in the hypoxic condition were collected and washed with cold PBS followed by lysis buffer. Harvested cells were lysed with RIPA buffer containing 1X protease inhibitor cocktail (Thermo Scientific, Rockford, IL) and centrifuged for 10 min at 10,000 rpm to collect the supernatants. The protein concentrations of the samples were determined with BCA protein assay kits (Thermo Scientific, Rockford, IL). Cell lysates containing 30 μg of protein were analyzed on 4–12% polyacrylamide gels (Life Technologies, Carlsbad, CA) and transferred to PVDF membranes, which were blocked in 5% non-fat dry milk (Biorad, USA) containing TBS-T (20 mM TRIS-HCl, pH 7.6; 150 mM NaCl; 0.1% Tween 20) (Fisher Scientific, Pittsburgh, PA) for 30 min at room temperature, then incubated with primary antibodies overnight at 4 °C followed by horseradish peroxidase (HRP)-labeled secondary antibodies (1:2000 dilution) in TBS-T for 2 h at room temperature. HIF-1α primary antibody was purchased from Novus Biologicals, USA, and CX3CR1 from R&D Biosystems, USA. Snail and matrix metalloproteinase (MMP)-9 were procured from Cell Signaling Technology (MA, USA). Signal detection was by electrochemiluminescence detection reagent (Fisher Scientific, Pittsburgh, PA). Protein bands were measured with Image Quant LAS4000 (GE Healthcare-Biosciences, Pittsburgh, PA).

### Quantitative reverse transcription polymerase chain reaction (qRT-PCR)

RNA isolation was accomplished according to our previous publication [28]. Briefly, cells were exposed for various times (3, 6, 9, or 12 h) to hypoxia and lysed with Trizol reagent (Invitrogen, Paisley, UK) followed by the standard protocol for RNA extraction. To mimic hypoxic conditions, cells were treated with CoCl_2_ (150 μM). RNA was precipitated and resuspended in nuclease-free water and quantified at 260 nm. cDNA was synthesized by use of 1 μg of RNA and reverse transcription super-mix for RT-qPCR reagent (Biorad, USA) following by the Bio-Rad PCR protocol. Primers for HIF-1α, CX3CR1, MMP-9, Snail, Twist, N-cadherin, and P-cadherin were synthesized with guidance from data in the National Center for Biotechnology Information gene bank database. The following sequences of the primers were used: HIF-1α: 5′-TTCCTTCTCTTCTCCGCGTG-3′ and 5′-ACTTATCTTTTTCTTGTCGTTCGC-3′; CX3CR1: 5′-AACCCCTGGAGGCGTTTAAG-3 and 5′-GATCCATGGTGAAGGCCCCA -3′; MMP-9: 5′- TTCAGGGAGACGCCCATTTC -3′ and 5′-AACCGAGTTGGAACCACGAC-3′; Snail: 5′-ATCGGAAGCCTAACTACAGC-3′ and 5′-CAGAGTCCCAGATGAGCATT-3′; Twist: 5′-AGCTGAGCAAGATTCAGACC-3 and 5′-CAGCTTGCCATCTTGGAGT-3′ N-cadherin; 5′-TACAGACATGGAAGGAATCCCC-3′ and 5′-ATGGCAGTAAACTCTGGAGGA-3′; and P-cadherin: 5′-CACCAACCATCATCCCGACA-3 and 5′-TCTGTGTTAGCCGCCTTCAG-3′. RT-PCR was performed by use of SYBR® Green PCR master mix reagents (Biorad, USA), and gene expression was analyzed by CFX-manager software (CFX96 Real-Time System-Biorad), 18S primer (5′-GGCCCTGTAATTGGAATGAGTC-3′ and 5′-CCAAGATCCAACTACGAGCTT-3′) was used as an endogenous control. The experiments were repeated three times.

### Migration and invasion assays

Migration and invasion assays were performed according to our previous publication [28]. Briefly, OVCAR-3, SW 626, and TOV-112D cells were grown, harvested, and counted (4 × 10^3^). To promote spheroid formation, they were seeded in 96-well spheroid formation plates at 37 °C. Culturex 96-well 3D Spheroid BME Cell Invasion Assay kits (Amsbio, MA, USA) were used. After spheroid formation (72 h), cells were incubated with invasion matrix and treated with CX3CL1 or a combination of CX3CL1 and KC7F2 (HIF-1α inhibitor). Then, cells were incubated for six days at 37 °C. To test the involvement of hypoxia in cell growth/proliferation, OvCa cells were treated with KC7F2 (40 μM) with CoCl_2_ (150 μM), and data were recorded for up to four days. At every 24 h, images were captured using the 10x objective of the microscope. Image-J software (NIH) was used to analyze the areas of migration and invasion.

### Statistical analysis

Statistical analysis was performed by deriving the standard error of means (±SEM) for at least three independent experiments. The level of significance was determined by one-way ANOVA, and *p*-values < 0.05 were considered as statistically significant.
